# A coherent family support model (Co-Fam) for preventing and treating childhood overweight and obesity: protocol for a randomized hybrid type 2 effectiveness-implementation trial

**DOI:** 10.1186/s12889-026-29530-3

**Published:** 2026-09-21

**Authors:** Ida Berdman, Anna Ek, Paulina Nowicka, Sara Raposo, Lydia Kwak, Markus Brissman, Susanne Andermo, Kristi Sidney Annerstedt, Liselotte Schäfer Elinder

**Affiliations:** 1 Department of Global Public Health, Karolinska Institutet, Solnavägen 1E, Stockholm, 113 65 Sweden; 2 Department of Clinical Science, Intervention and Technology, Karolinska Institutet, Stockholm, Sweden; 3 Department of Food Studies, Uppsala University, Uppsala, Sweden; 4 Institute of Environmental Medicine, Karolinska Institutet, Stockholm, Sweden; 5 Department of Neurobiology, Care Sciences and Society, Karolinska Institutet, Stockholm, Sweden; 6 Department of Physical Activity and Health, The Swedish School of Sport and Health Sciences, Stockholm, Sweden; 7 Centre for Epidemiology and Community Medicine, Region Stockholm, Stockholm, Sweden

**Keywords:** Children, Family support, Parental support, Prevention, Obesity treatment, Randomized control trial

## Abstract

**Background:**

Swedish child healthcare involves school health services, primary healthcare, and pediatric clinics. Family-based programs addressing child diet, eating behavior, parental feeding practices and physical activity show promise for preventing and treating overweight in children 2–12 years, but organizational gaps hinder coherent implementation of interventions. The novel Co-Fam model integrates two complementary programs: the universal Healthy School Start (HSS) family support program, delivered in schools during grade 1, and the targeted 12 months More and Less (ML) parental support program, provided through primary healthcare to treat children 6–12 years with overweight and obesity. ML consists of ten group sessions with parents, followed by individual booster sessions. The dual primary aim of this study is to evaluate the effectiveness of the ML program on child bodyweight development and to assess implementation outcomes fidelity, acceptability, feasibility and cost-effectiveness of the Co-Fam model.

**Methods:**

The setting for this study is a municipality in Region Stockholm with high cultural diversity and health needs, which has been running the HSS program since 2022. In this hybrid type 2 effectiveness-implementation study with two parallel arms, children aged 6–12 years with overweight or obesity will be randomized to either the ML program or standard care. Primary effectiveness outcomes are BMI z-score and %IOTF-25 measured at baseline, three and 12 months. Secondary outcomes are waist-to-height-ratio, parental feeding practices, child physical activity, diet and eating behavior. Primary implementation outcomes are fidelity to the ML program assessed at three and 12 months. Acceptability and feasibility of the Co-Fam model will be assessed three to 12 months after start, through semi-structured interviews with parents and providers. A health economic evaluation will be conducted using a cost-utility design.

**Discussion:**

We hypothesize that ML will be more effective than standard care in reducing overweight and obesity in children. The integration of the HSS and ML programs in the Co-Fam model is expected to enhance collaboration between school and healthcare and ensure early identification of at-risk families. The dual focus on effectiveness and implementation will inform the scalability and sustainability of the Co-Fam model with implications for policy and practice in child obesity prevention and management.

**Trial registration:**

The trial was registered at Clinicaltrials.gov, September 11-2025, ID: NCT07158944.

**Supplementary Information:**

The online version contains supplementary material available at https://doi.org/10.1186/s12889-026-29530-3.

## Background

Reducing overweight and obesity in children is a global public health priority [1]. In Sweden, about one in five children aged 6–9 years have overweight (15%) or obesity (6%) [2]. The prevalence is rising across all population groups, with a pronounced social gradient, disproportionately affecting individuals with lower educational attainment, low income, single parent households [3], and individuals born outside of Europe [4]. The development of obesity is complex, influenced by genetic predisposition, environmental and social conditions as well as health related behaviors such as physical activity and dietary habits [5]. Children living with obesity face an increased risk of cardiovascular morbidity [5] and type 2 diabetes (T2D) in adulthood [6, 7]. In addition, individuals with overweight or obesity often experience weight-related stigma, which can lead to psychological distress, including low self-esteem, depressive symptoms, and anxiety, and may contribute to unhealthy coping strategies and avoidance of healthcare [8]. Fortunately, evidence based person-centered pediatric obesity treatment can help reduce these risks, improving long-term health outcomes by lowering the likelihood of T2D, dyslipidemia, and hypertension, and even decreasing mortality rates in young adulthood [9].

Overweight and obesity impose substantial lifetime societal costs, primarily due to elevated healthcare expenditures and productivity losses [10]. More specifically, the average incremental lifetime healthcare cost for a 6-year-old child with obesity in Sweden, compared to a child with normal weight, is estimated at approximately €39 270 for girls and €23 741 for boys [10]. For children with overweight, the corresponding cost is around €13,700 for both sexes [10]. Furthermore, children with a high body mass index (BMI) experience increased lifetime productivity losses later in life due to temporary and permanent sick leave, as well as increased risk of unemployment [10]. Around 50% of children who have developed obesity before puberty will continue to live with obesity during adolescence, and of these, around 80% will have obesity in adulthood [11]. Hence, the complexity related to obesity development calls for a dual approach of prevention and management including *universal* components for all children such as promoting healthy eating habits and physical activity in healthy school and home environments [12] as well as *targeted* interventions for high-risk children focusing on behavior change [13].

Family-based interventions involving parents only, alternatively parents and children, are recommended for obesity prevention for children up to 12 years of age, since parents influence and to a large extent have control over their child’s environment [14–17]. Family-based interventions can improve child weight and/or weight-related behaviors [17, 18]. Interventions often focus on diet and physical activity, sometimes including screen time and sleep. They are usually provided by primary healthcare (PHC), schools, or other actors in the community [15, 16]. Effective interventions focus on parenting skills and practices, role modelling and strategies for managing children’s behavior, such as reinforcement [17], grounded in social learning theory [19]. Systematic reviews further recommend multi-component behavior change interventions to prevent and manage childhood obesity [18, 20, 21]. While school-based interventions show effectiveness regarding prevention [22], family-based interventions are effective in reducing the BMI of children with obesity [18, 20]. However, the effectiveness of interventions depends not only on parental involvement but also on broader contextual factors, including family functioning, cultural and socioeconomic circumstances, and access to resources [18]. The largest reductions in BMI z-score have been observed in younger children, particularly those aged 2–5 years, followed by children aged 6–11 years when participating in comprehensive, multicomponent interventions for overweight and obesity [21]. It is generally recommended that family-based interventions should last at least six months [23].

In Sweden, national guidelines for the treatment of children with obesity recommend family support and combined health behavior treatment, including at least 26 contact hours with a multidisciplinary team with child competence [24–26]. The type of treatment, number of visits and overall intensity should be tailored by the clinician to individual needs, age, degree of obesity and any comorbidities. While some regions in Sweden offer either individual or group-based parental support, these approaches have not been systematically evaluated for children aged 6–12 years.

Based on the Exploration, Preparation, Implementation, Sustainment (EPIS) framework [27], we developed the Co-Fam model together with school health services, PHC including dieticians and physiotherapists, a pediatric clinic (PC) and parents to increase acceptability and feasibility [28, 29]. Obesity treatment for children in Sweden involves a collaboration between schools run by municipalities or private corporations, and healthcare run by regional authorities [30], all publicly funded. Implementation of interventions in multi-organizational settings needs careful planning to be successful. The Co-Fam model combines the universal “Healthy School Start” (HSS) family support program delivered by school personnel in grade one with a focus on health promotion and prevention with the targeted “More and Less” (ML) obesity treatment program offering parental group sessions by PHC personnel. The HSS program has been evaluated for children aged 5–7 in three cluster-randomized controlled trials with beneficial effects on diet [31–33], and a significant decrease in BMI in children with obesity [12]. The more intense and targeted ML program for parents of children aged 2 to 6 years with overweight or obesity has shown clinical effectiveness in two randomized controlled trials [13, 34, 35]. In order to maintain and enhance the weight change seen in the HSS program for children with higher needs, the ML program could be effective. Accordingly, the Co-Fam model builds on a combination of the HSS and ML program. This requires adaptation of ML for parents of children aged 6 to 12, for whom evidence-based parental support is not available in Sweden. The Co-Fam model is novel in the since that it integrates these two programs across settings and levels of care, creating a coherent pathway from universal prevention to targeted treatment according to child needs.

We hypothesize that combining the HSS and ML programs in a coherent model will be feasible and acceptable, increase health literacy, ensure early identification of families in need, and enable early treatment of overweight and obesity. In addition, we hypothesize that it will lead to better collaboration between school health services, primary and specialized healthcare, ultimately improving prevention and treatment of children with overweight and obesity. This study aims to evaluate both the effectiveness of the ML program and implementation of the Co-Fam model in the Swedish context.

The specific research questions are:

1. What is the effectiveness of the 12-month ML program on children’s weight development, compared to standard care for overweight and obesity in 6 to12 year-old children?
2. What are the effects of the ML program on children’s diet, eating behavior, physical activity and on parental feeding practices, compared to standard care?
3. Is the Co-Fam model acceptable, feasible, and cost-effective?

## Methods

### Co-design and public involvement

Phase 1 of the Co-Fam project, the exploration and preparation phase of the EPIS framework [27], involved a co-design process in which personnel from school health services, PHC, PC, and parents participated in workshops. The workshops aimed to identify barriers and facilitators, and to develop and tailor the implementation strategies to the context to establish high acceptability. This protocol paper describes phase 2 of the Co-Fam project, the implementation phase.

### Study design

In this hybrid type 2 effectiveness-implementation study [36] with two parallel arms, children aged 6–12 years with overweight or obesity will be randomized to either the ML program or standard care. The universal HSS program has been running in primary schools in the municipality since 2022. The intervention arm will receive the ML program adapted to children 6–12 years by the researchers who developed the program who are part of the research team (PN and AE). The control arm will receive standard care for overweight or obesity. Both arms are followed over a period of 12 months. Personnel from school health services, PHC, and PC will be involved in recruitment, implementation and follow-up. PHC personnel, mainly dieticians and physiotherapists, have been trained as group leaders and will deliver the ML program to parents.

The study will be conducted in line with the ethical principles for medical research involving human subjects in the Declaration of Helsinki [37]. This protocol was developed in accordance with the

Standard Protocol Items: Recommendations for Intervention Trials (SPIRIT) guidelines [38]. The intervention is reported using the template for intervention description and replication (TIDieR) [39] checklist. The guidelines are presented in Supplementary Table 1.

### Study setting

The study will take place in a municipality with 19 public primary schools with a prevalence of childhood obesity among the highest in the Stockholm region [40]. Based on routine data it is estimated that approximately 1,800 children aged 6–12 years in this municipality are living with overweight or obesity, some of whom receive care in the primary or specialist setting. The municipality has just over 100,000 inhabitants out of which more than 44% are born outside of Sweden, twice as many as the national average [41].

In Sweden, access to school health services is mandatory and regulated by law [42]. National guidelines mandate that school health services offer health checks for children on at least three occasions during grade one to six (age 6–12 years) including at least two measurements of height and weight [42]. Children classified as having overweight according to International Obesity Task Force (IOTF) criteria [43] are entitled to support from PHC while children classified as having obesity are entitled to treatment from specialized pediatric care [25]. If a school nurse identifies a child with obesity, a referral is sent to a pediatric clinic (PC) after informing the parents. In case of overweight, a referral is usually sent to PHC in agreement with the child’s parents for a meeting with a dietitian or physiotherapist [25]. Table 1 includes a detailed description of the guidelines. School health services, PHC and PCs can be under public or private governance, but all are publicly funded. This creates challenges for communication and coordination between different levels of care, as the use of separate medical record systems means that school health services often lack easy access to information about a child’s treatment in primary and specialist care [30]. The Co-Fam model has been designed to bridge this gap and to ensure coherent support according to needs.

**Table 1:** National and regional recommendations for management of overweight and obesity for children up to 12 years

	Overweight	Obesity
Provider	School and Primary healthcare	Specialized pediatric clinic in collaboration with primary healthcare
Diagnosis	Growth curve (BMI z-score) and assessment of health behaviors in the family	Growth curve (BMI z-score), assessment of health behaviors in the family, blood samples of the child to check for co-morbidities
Recommended treatment	Provide basic advice on nutrition and physical activity Offer individual or family-based support for children with unhealthy eating habits and/or insufficient physical activity, including: -Counseling -Motivating strategies -Behavior change strategies -Positive reinforcement -Parental support Identification of children at risk of developing obesity, refer children who develop obesity to a pediatric clinic	Combined health behavior treatment -Individual and parental support with healthy eating habits, physical activity and sedentary behavior -Support for behavior change regarding sleep, stress and self-regulation -Long-term support to maintain behavior change At least 6 visits per year including a yearly check-up with a pediatrician and contact with a dietician and physiotherapist A recommendation of at least 26 h of treatment per year Parental support programs and digital support are offered depending on availability and interest In case of severe obesity with complications, families can be referred to the National Centre for Child Obesity. Medications can be prescribed as a complement to combined health behavior treatment (if child is > 12 years)
References	[14]	[25, 44]

### The Co-Fam model

**Fig. 1:**
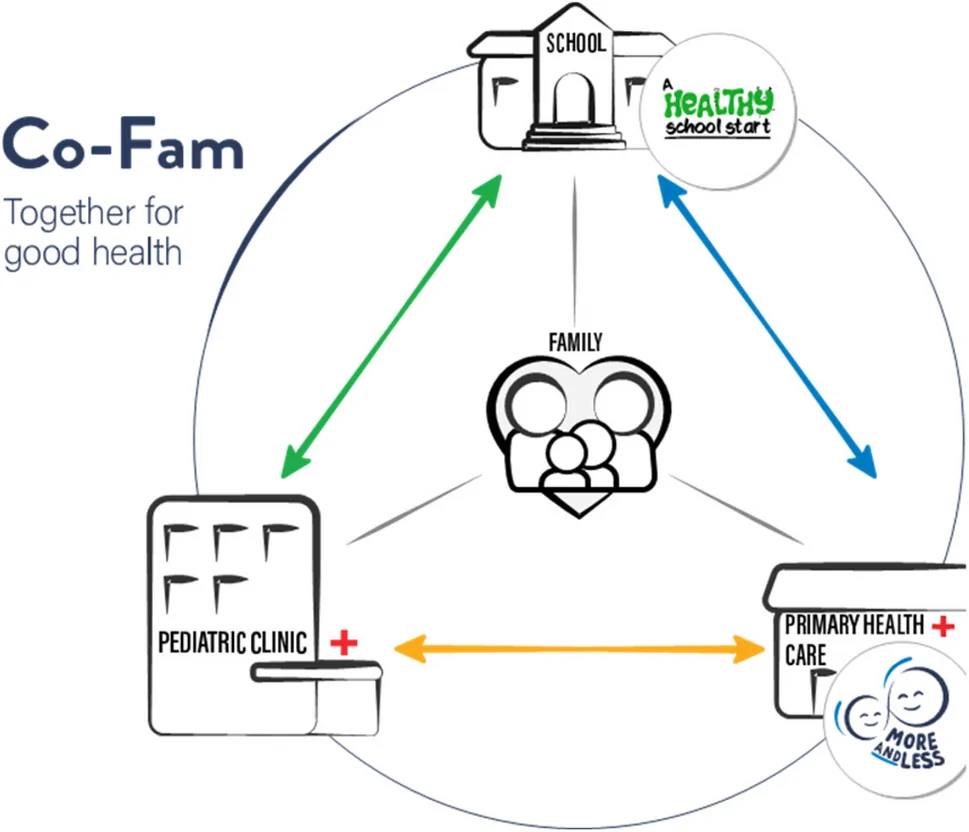
The Co-Fam model

The Co-Fam model (Fig. 1) illustrates a coherent model of family support, combining the universal HSS and the targeted ML program. Together, the two programs aim to promote healthy habits and positive parenting practices to prevent and treat overweight and obesity in children. The model builds on a close collaboration between families, school health services, PHC and PC, recognizing the importance of each actor in supporting the child´s development and maintaining a holistic perspective with the family in the center. The arrows represent referrals of children diagnosed with overweight or obesity between school health services, PHC and PC. The arrow between PHC and PC indicates collaboration in offering treatment options for families with higher needs.

The HSS program has been provided in grade one to all children and their families attending public schools in this municipality since 2022. The program is based on Social Cognitive Theory [19] and consists of four components: (1) A parental guide with health information available in 11 languages; (2) a motivational interview with parents performed by the school nurse; (3) classroom activities for the children performed by teachers with home assignments; and (4) a T2D risk self-assessment test for parents [44]. For a detailed description of the HSS program see Elinder et al. [45]. The HSS program has been evaluated regarding implementation showing that the program can be performed with relatively high fidelity, although somewhat lower in families with a non-Nordic background mainly due to language issues when performing the motivational interview [46]. Qualitative studies with school personnel and families have shown that teachers, school nurses, parents and children overall have a positive attitude towards the program and that it has raised the health literacy of the participants in both theory and practice [47–50].

The ML parental support program is grounded in Social Learning Theory [51, 52] and Social Interaction Learning Theory [19, 51] which together emphasize how parenting practices, social interactions, and broader environmental contexts influence children’s behaviors [53]. The ML program is based on a manual and consists of ten weekly 90-minute group sessions, led by primary healthcare personnel (HCP) (dieticians and physiotherapists) trained as group leaders (Table 2). The group sessions are followed by six individual booster sessions every six weeks for another nine months. Group sessions focus on strengthening parent-child communication to achieve healthy behaviors through evidence-based parenting practices, including positive reinforcement and role modeling, with role play and peer discussions as key components for building skills. Parents receive handouts briefly outlining the content of each session, including home assignments. The booster sessions focus on the families’ individual needs and provide an opportunity to follow up on the relevant topics covered during the group sessions.

**Table 2:** Content of the group sessions in the More and Less parental support program (month 1–3)

Session	Topic	Content overview
1	Introduction and parents’ key roles	Presentations, aim of program, parents’ key roles and why treatment of obesity.
2	When More and when Less?	Tools for healthy eating, screen-time, physical activity, and sleep.
3	Cooperation and encouragement	Importance of cooperation and encouragement in the family. What is an appropriate portion size.
4	Teaching new behaviors	Encouragement and incentives. Setting up house rules. Being aware of the influence of social media.
5	Using charts and incentive systems	Using incentive systems to learn new behaviors. Promoting body satisfaction and positive self-esteem in the child.
6	Parents as teachers: To prepare and to plan	How to decrease conflicts. Healthy food choices – the Keyhole symbol and snack meals.
7	Setting limits	To set limits - why and how? The plate model.
8	Power-struggles	Avoiding and disengaging from power struggles. Keeping emotions under control. Improving recipes.
9	More support Less stress	How to handle stress and involve their network. Promote mental health.
10	Summary and preparation for the future	Lessons learned and moving forward. Promote social contacts.

### Intervention arm: the ML program

Families in the intervention arm will receive the 12-month ML group-based program. Over the study period (January 2026 – December 2028), two ML groups will be initiated every six months, each including 6–10 parents. Group sessions will be offered weekly, either face-to-face at PHC clinics or online, while booster sessions will be provided by phone or videocall. If a parent misses a session, a follow-up call is offered. If a parent does not attend any of the first three sessions, they will be invited to join the next available group in the study.

### Control arm: standard care for overweight or obesity

**Fig. 2:**
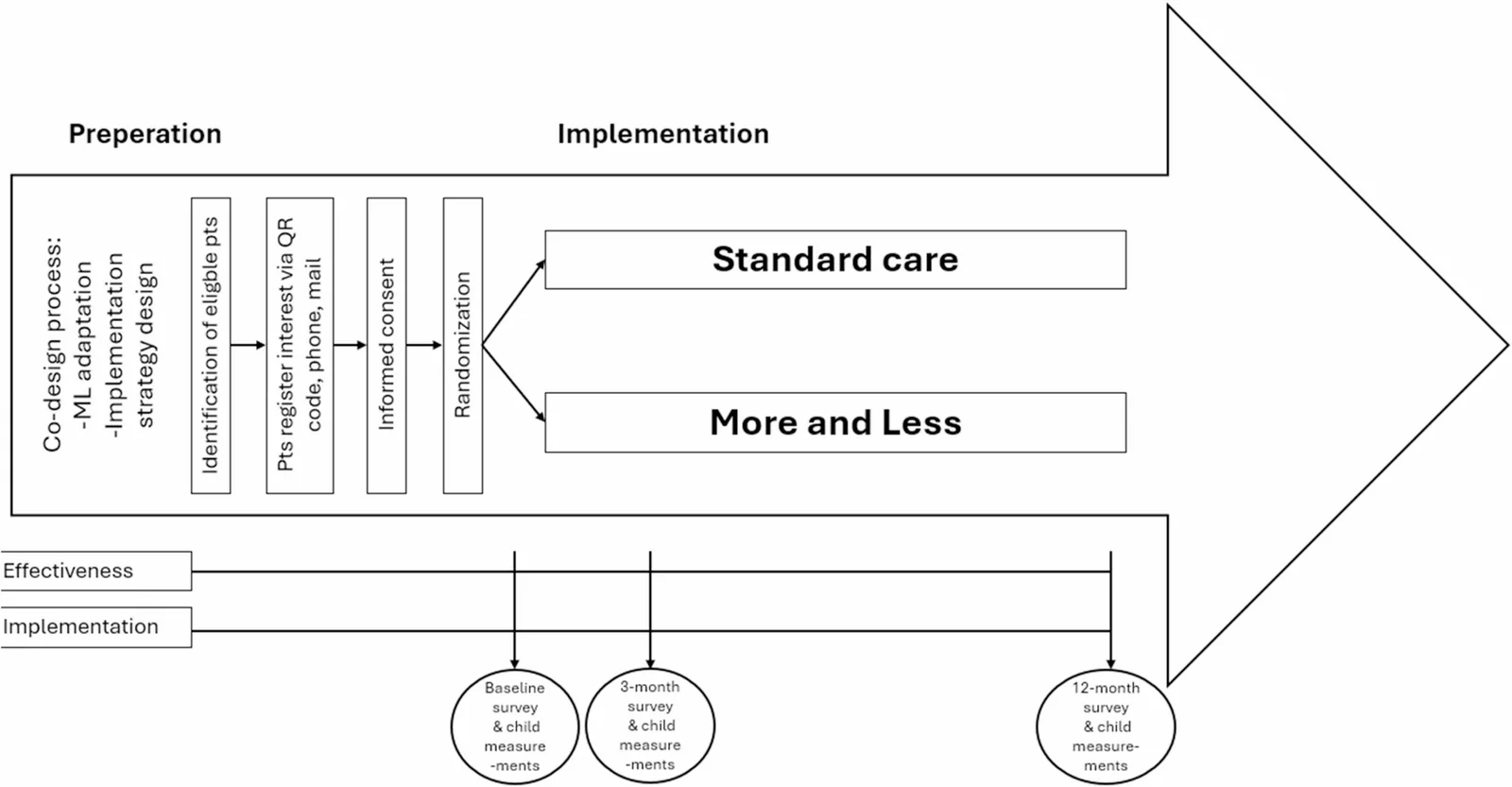
Study timeline. Abbreviations: pt; participant

Standard care for overweight [54] and obesity [25] is based on national guidelines (Table 1) where children with obesity are referred to a pediatrician for yearly check-ups often in combination with follow-up visits to a pediatric nurse, dietician and/or physiotherapist. For some children, referral to a psychologist can also be part of obesity treatment. They also have the option to receive a digital support tool. For children with overweight, parents are offered referral to the PHC for support by a dietitian and physiotherapist. All children’s weight, height, and waist circumference will be measured on three occasions (Fig. 2). A timeline of the study procedures is illustrated in Fig. 2.

### Register-based control group

In addition to the randomized controls, data from the Swedish National Child Obesity Treatment Register BORIS, started in 2005 [55], will be collected retrospectively to enable a national comparison of the effect of standard treatment to the ML program for children with obesity. Data from children in the BORIS registry will be selected randomly in a 1:3 ratio matched to children with obesity in the intervention arm on age (± 100 days), sex, BMI z-score at baseline and year of treatment initiation. The type of care received will be documented retrospectively over a period of 12 months based on registry data.

### Recruitment of participants to the trial

The school health services will identify children aged 6–12 years with overweight and obesity during routine health and growth checks and inform the parents about the ongoing study and possibility of participating. Likewise, PHC and PC can recruit eligible participants to the trial. A conditional incentive (gift voucher worth €85) will be provided after the end of the study to reimburse parents for the time they spent on study-related activities. A written informed consent will be obtained from all parents by a research assistant via REDCap or via post and prepaid envelopes. Written consent will be obtained from both parents when applicable, even if only one of them participates in the study. Children will be informed about the study by their parent(s) and asked whether they agree to the collection of height, weight and waist measurements. Participation is voluntary, and parents will be informed that they can withdraw at any time without explanation.

### Inclusion and exclusion criteria for the trial

Eligible participants are parents of children between 6 and 12 years of age who have children with overweight or obesity according to IOTF criteria [43]. Children taking medication affecting weight development will be excluded from the study. Families in the intervention group are advised not to participate in other forms of structured treatment during the study period. The ML group sessions will be conducted in easy Swedish and potentially in other languages depending on needs.

### Sample size

We estimate that the trial will need 126 children (63 children in the intervention and 63 in the control group) to be able to detect a difference at 12 months between the groups in BMI z-score of -0.16 with a standard deviation of 0.3, 80% power and an alpha level of 0.05, including an estimated 10% loss to follow-up.

### Implementation of the Co-Fam model

**Fig. 3:**
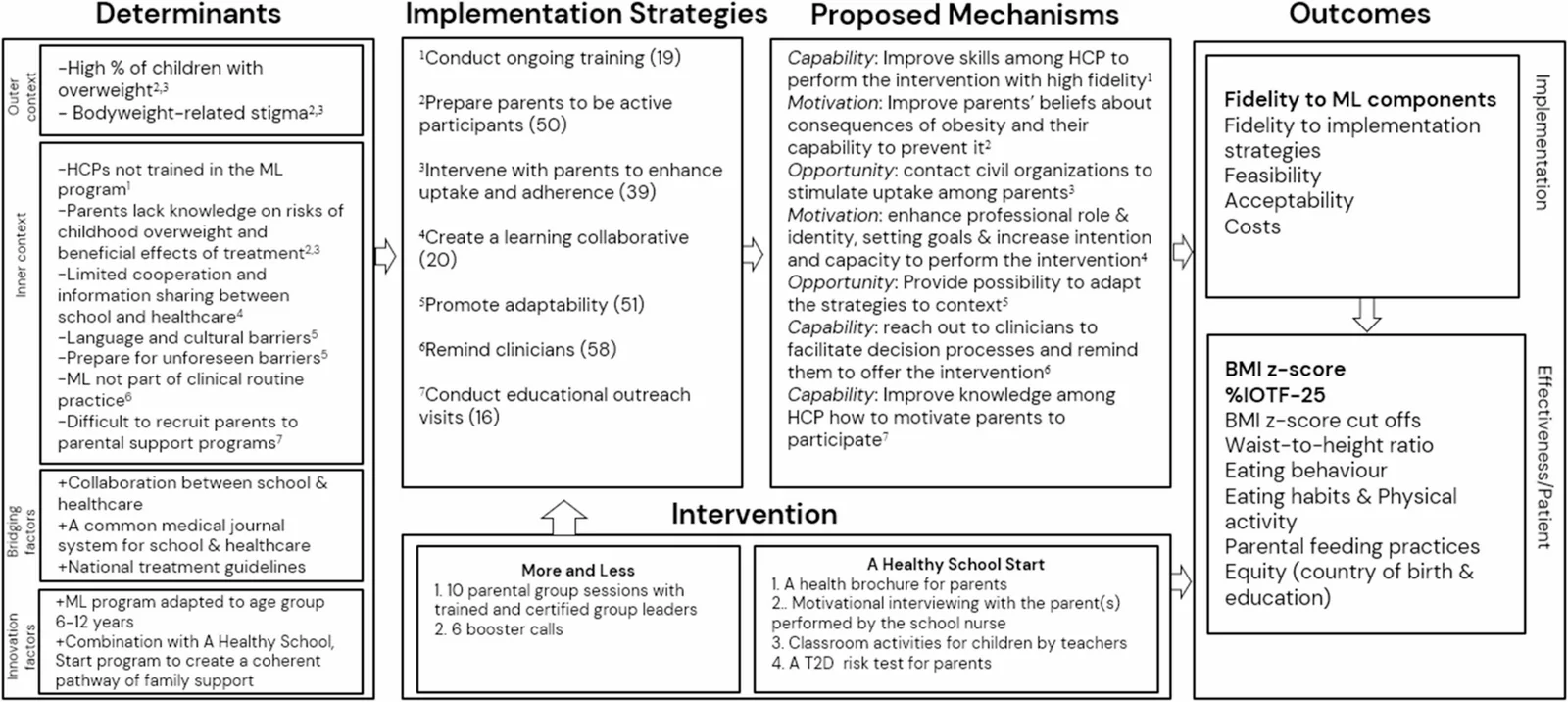
Implementation Research Logic Model. Determinants are marked as a barrier (-) or facilitator (+). Strategies are linked to determinants and mechanisms through footnotes. Mechanisms are based on COM-B and the Theoretical Domains framework. Abbreviations: ML, More and Less; HCP, Health Care Personnel; BMI, Body Mass Index; IOTF, International Obesity Task Force, T2D, Type 2 diabetes

An Implementation Research Logic Model (IRLM) [56] was developed during the exploration and preparation phase through a co-design process involving parents and personnel from PHC, PC and school health services, conducted in a series of workshops. This process is described in detail in a separate paper [29]. The hypothesized relations between determinants, strategies and mechanisms are shown in Fig. 3. Implementation strategies based on the Expert Recommendation for Implementing Change (ERIC) taxonomy [57, 58] and their form and function [59] were discussed and approved by HCP to ensure contextual and clinical feasibility. The IRLM includes determinants in the outer context, inner context, bridging factors and innovative factors based on the EPIS framework [27]. Mechanisms of change of the strategies were hypothesized based on the Capability, Opportunity, Motivation and Behaviour (COM-B) model and the Theoretical Domains Framework (TDF) [60]. Outcomes refer to effectiveness of the ML program and of implementation of the Co-Fam model. The HSS components and related implementation strategies have been evaluated previously [46].

### The implementation strategies

The strategy *Conduct ongoing training* is a four-day course for HCPs in the ML program based on a manual, including supervision and feedback. The strategy *Prepare parents to be active participants* includes HCPs providing information to families meeting the inclusion criteria about consequences of overweight in children and the ML program. The strategy *Intervene with parents to enhance uptake and adherence* includes HCPs distributing information leaflets in different languages to support recruitment and contact with civil organizations within the community to increase uptake of hard-to-reach groups. The strategy *Create a learning collaborative* includes meetings of the research team together with HCPs from PHC and school health services every six months to discuss study progress and potential problems. The strategy *Promote adaptability* enables revision of strategies if needed (while keeping the intervention components intact), to be agreed on during the learning collaborative meetings. The strategies *Remind clinicians* and *Conduct educational outreach visits* aim to support HCPs during implementation. Reminders include newsletters sent four times per year with updates about the study and useful tips for recruitment and meetings with families. Outreach visits include inspirational lectures for HCP on two occasions per year. Implementation strategies are described in more detail elsewhere [29].

### Randomization

When parents have signed the consent form, they will be randomized to a treatment arm using REDCap randomization module [61]. A block randomization with a block size of six will be used to ensure a balanced allocation across treatment arms within each stratum. Stratification into the two strata overweight and obesity will be performed based on the baseline BMI z-score. Each block of six will have three participants in the intervention arm and three in the control arm, and the order of assignments within each block will be random. To include 126 participants, 21 blocks are needed. A research assistant will inform the clinics about which arm the participant belongs to. Neither HCPs nor parents will be blinded to allocation. The statistician remains blinded to group allocation until the analyses are performed to ensure objectivity. HCPs will not have access to the random allocation sequence.

### Primary outcomes

The primary outcomes, BMI z-score and %IOTF-25, will be assessed at baseline, after three and 12 months for both arms. The BMI z-score is calculated based on age- and sex-specific IOTF reference data [43]. The %IOTF-25 is a percentage-based index of children above the IOTF threshold for overweight. Combining it with the BMI z-score may be better suited to follow changes in weight for children and adolescents with obesity [62].

The primary implementation outcome is fidelity to the ML program. Dose delivered is measured by number of ML group sessions and booster sessions delivered while dose received is measured by average number of ML group sessions and booster sessions received per participant. Adaptations made to the sessions during the trial and reasons for adaptations will be documented by asking ML group leaders after each completed group. Efforts to increase program fidelity include supervision of ML group leaders during their first ML group by the trainers. Participant responsiveness will be evaluated through questionnaires at week five and ten of the ML program and through interviews after the ML program is finalized.

### Secondary outcomes

The probability of reaching a reduction in BMI z-score cut-offs of ≥ 0.25 or ≥ 0.5 will be used as they indicate a clinically significant reduction in BMI [63]. Waist-to-height ratio (WHtR) will be compared between groups to estimate excess adiposity [64]. The child’s dietary habits will be assessed through the validated Eating and Physical Activity Questionnaire (EPAQ) [65] using nine questions on dietary habits. Parents are asked to recall the child’s intake of unhealthy and healthy foods and unhealthy drinks. Questions are answered as amounts of each item (deciliters, glasses or portions) from 0 to 7 or more. The child’s physical activity level will be assessed through three questions asked to the parents [66]. Questions are answered by estimating the number of days of physical activity per week (0–7), number of times of exercise per week (0–7) and the proportion of time spent sedentary during the week (0–5). The child’s eating behavior will be assessed using the validated Children’s Eating Behavior Questionnaire (CEBQ) [67] answered by a parent. It consists of 35 questions on eight scales that identify behavioral traits that may contribute to obesity or disordered eating. Parents rate their child’s eating behavior on a five-point Likert scale (never, rarely, sometimes, often, always). Parental feeding practices will be measured using the validated Comprehensive Parental Feeding Questionnaire (CPFQ) [68, 69] including five factors: Monitoring of children’s food intake, pressure to eat, restriction of food, use of food for emotional regulation, and healthy eating guidance. It is comprised of 36 questions in which parents rate their feeding practices on a four-point Likert scale (rarely, sometimes, often, very often) or (disagree, somewhat disagree, somewhat agree, agree). Potential adverse events could be rapid weight loss, sudden unhealthy change in eating behavior and/or physical activity. This will be monitored continuously and affected families will be guided to appropriate care.

Fidelity to implementation strategies will be assessed as described in Table 3. Potential adaptations to implementation strategies should support sustainability to ensure that the ML program can be integrated into routine practices when the study is commenced [70, 71]. If any adaptations to implementation strategies are suggested by the HCPs, the Model for Adaptation Design and Impact (MADI) will be used to decide if an adaptation is needed and evaluate the adaptation. MADI involves a description of what is modified, nature of adaptation, who participated in adaptation decision-making, for whom the adaptation was made, and when the adaptation occurred [71].

**Table 3:** Assessment of fidelity to the implementation strategies based on a checklist, survey and interviews

Strategy	Fidelity dimension	Assessment	Method/ Performed by
Conduct ongoing training	Adherence	Number of training opportunities offered to HCP and number of participants in ML training	Checklist by researchers
	Responsiveness	Survey at the end of the ML training	Survey to ML group leaders
Prepare parents to be active participants	Adherence	Number and type of activities performed by HCP to motivate parents to be active in ML sessions	Checklist by researchers, survey to HCP
	Responsiveness	Individual interviews with parents about their motivation to participate and school nurses about the referral process	Interviews by researchers
Intervene with parents to enhance uptake & adherence	Adherence	Number and type of activities performed in the community to motivate parents to be active	Checklist by researchers
	Responsiveness	Individual interviews with parents, school nurses and civil organizations in the community about uptake and engagement	Interviews by researchers
Create a learning collaborative	Adherence	Number of learning collaborative meetings for HCP and number of participants	Checklist by researchers
	Responsiveness	Interviews with healthcare personnel about the usefulness of the collaborative meetings	Interviews by researchers
Promote adaptability	Adherence	Number of adaptations requested, evaluated and made	Checklist by researchers
	Responsiveness	Interviews with healthcare personnel evaluating potential adaptations	Interviews by researchers
Remind clinicians	Adherence	Number of newsletters sent to HCP	Checklist by researchers
	Responsiveness	Individual interviews with HCP about the content of the reminders and their usefulness	Interviews by researchers
Conduct educational outreach visits	Adherence	Number of outreach visits and attendance	Checklist by researchers
	Responsiveness	Individual interviews with healthcare personnel about the usefulness, content and appreciation of the outreach visits	Interviews by researchers

*Abbreviations:HCP* Healthcare personnel, *ML* More and Less

Acceptability is the perception among stakeholders that a given treatment, service, practice, or innovation is agreeable, palatable, or satisfactory [72], while feasibility is the extent to which a new treatment or an innovation can be successfully used or carried out in a given setting [72]. Acceptability and feasibility will be evaluated with individual interviews with approximately two to three parents from each ML group (total ~ 16 parents) three to 12-months after start, and with providers at the end of the 24-month follow-up.

A health economic evaluation of the Co-Fam model will be conducted using a cost-utility design, applying a decision tree and Markov model over a lifetime horizon. The model will be driven by changes in BMI resulting from the trial. Costs will be obtained from the Co-Fam model and the ML program, supplemented with data from the literature and other publicly available sources. Additional parameters, including transition probabilities and utility weights for different health statuses will be derived from both the literature and intervention data. Quality-adjusted life years (QALY) will serve as the measure for utility. Cost-effectiveness will be expressed as an incremental cost-effectiveness ratio (ICER). Sensitivity analyses will be performed to assess the robustness of the results to plausible variations in key parameters and assumptions.

### Data collection

Data collection activities, time points, and data sources are shown in Table 4. Children’s height, weight, and waist circumference will be measured by the school nurse at three timepoints. Parents will answer a baseline questionnaire including demographic data (sex, age, country of birth, years in Sweden, education, housing situation, employment status, weight, height, number of children) and their child (sex, age, country of birth, school, treatment clinic, additional diagnoses). Questionnaires regarding the child’s diet and physical activity habits, the child’s eating behavior, and parental feeding practices are included in the three assessments. Questionnaires can be answered from a mobile phone/tablet/computer, at a convenient time for the participant. Data regarding number of treatment visits and comorbidities will be collected from the child’s medical journal. Data on implementation outcomes will be collected from parents and HCPs via questionnaires distributed during the study period and semi-structured interviews performed after participation in the ML program. Data from the BORIS registry [55] will be collected retrospectively.

**Table 4:** Data collection and time points

TIMEPOINT	Enrollment and randomization	TRIAL PERIOD		
	Pre-intervention	T0	T1	T2
ENROLLMENT:	X			
Eligibility screen	X			
Informed consent	X			
Randomization	X			
INTERVENTION/COMPARATOR:				
* Intervention: More and Less*		X	X	X
* Comparator: Standard care*		X	X	X
* Comparator: Register control*		X	X	X
ASSESSMENTS: Effectiveness outcomes				
* Child’s eating habits*		X	X	X
* Child’s physical activity*		X	X	X
* Child’s eating behavior*		X	X	X
* Parental feeding practices*		X	X	X
* Child’s weight*,* height and waist*		X	X	X
ASSESSMENTS				
* Demographics*		X		
* Adverse events*			X*	X*
* Fidelity*			X	X
* Feasibility*				X
* Acceptability*				X
* Cost*				X

*Data on adverse events is collected continuously. Abbreviations: T0, baseline; T1, 3-month follow-up, T2, 12-month follow-up

### Data management

A data management plan has been developed using the Swedish Research Council template including plans for secure storage and data quality. All participants will receive a personal code and data will be pseudonymized. Data will be collected and managed using the electronic data capture tool REDCap [61] on secure, encrypted servers at Karolinska Institutet with access restricted to authorized study personnel. Regular backups will be performed, and an audit trail will document all data changes. Qualitative data will be recorded and transcribed verbatim, and interviews will be pseudonymized before analysis. All data will be retained for 10 years after study completion in accordance with GDPR and institutional policies, after which it will be securely destroyed. The findings will be disseminated through peer-reviewed open-access publications, presentations in the municipality and reports for stakeholders and end-users.

### Data analysis

All analyses will be performed as an intention-to-treat analysis. Descriptive statistics will be used to summarize participant characteristics at baseline per study arm. Prior to analysis, the distribution of the outcomes will be assessed for normality and homogeneity. Between arm differences for outcomes, BMI z-score (primary outcome), %IOTF-25, probability of reaching BMI z-score cut-offs and WHtR will be analyzed.

To analyse the primary outcome of BMI z-score we will fit a linear mixed model with BMI z-score at 0, 3, and 12 months as the outcome variable, including a random intercept for each child. Fixed covariates will be treatment assignment (ML or control), time (in months), their interaction, and baseline weight status. The effect of the treatment will be assessed by the interaction parameter, which is interpreted as the difference in change of BMI z-score per month” between the two randomisation arms. A dose-effect analysis will be performed to assess the effect of number of ML sessions attended. This will be performed by replacing the treatment variable in the regression model with a variable indicating how many sessions the child’s parents attended.

The register control arm will be compared against the ML arm regarding BMI z-score, %IOTF-25, probability of reaching BMI z-score cut-offs and WHtR, using the same methods as described above adjusted for confounders. Secondary outcomes will be analyzed using the same principles as above. Interviews with parents and HCP will be transcribed verbatim and analyzed using reflexive thematic analysis [73–75]. We will explore causal pathways between effectiveness- and implementation outcomes by integrating the quantitative and qualitative data. Missing data will be evaluated for its extent and patterns. If missingness in key outcomes (e.g., BMI z-score, %IOTF-25, BMI z-score cut-offs, WHtR) exceeds a predefined threshold, appropriate statistical methods such as multiple imputation will be applied. Full details about the analysis will be described in the Statistical Analysis Plan, which will be finalized and published in Clinicaltrials.gov before the end of data collection.

## Discussion

The Co-Fam model combines two evidence-based programs that align with the recommended components for supporting families with children aged 6–12 years, namely being theory-based, focusing on parenting practices and role modelling, and using a person-centered approach, as outlined in the national guidelines for treatment of overweight and obesity in children [25]. Parents exert a key influence on the home-food environment and on the opportunities available for children to be physically active. Thus, support for parents to facilitate healthy habits needs to be strengthened, especially for parents born outside of the Nordic region whose children have a higher prevalence of overweight and obesity than Nordic-born [4]. Furthermore, targeting children before entering puberty has been found to be more effective on future weight development than in older children [23]. Since structured evidence-based parental support programs for children 6–12 years with overweight and obesity are lacking in Swedish healthcare today, many families do not get the recommended support. Thus, if shown to be feasible and effective, the Co-Fam model will provide an evidence-based approach to preventing and treating unhealthy behaviors and overweight and obesity in children [25].

Families with children attending public school in the municipality automatically receive the HSS program, which is integrated into the school’s curriculum and has been successfully delivered since 2022. As the program is delivered during regular classes, families cannot opt out. However, for participation in the ML program, both parents and the child must provide consent. Both parents are encouraged to participate to support consistency in parenting practices. Children will be informed about the study by their parents and asked to consent to the measurements of height, weight and waist circumference which are taken by the school nurse. School nurses are responsible for student health and routinely perform these measurements as part of national follow-ups. Therefore, collecting measurements in this context is considered the least intrusive approach.

A strength of the Co-Fam model, if effective and feasible, will be a closer collaboration between schools under the municipal governance and PHC and PC under regional governance, bridging the organizational gap between these actors and leading to early interventions for all children. A limitation is the time commitment required by the ML program, comprising ten group sessions and subsequent booster sessions, which will impose challenges in recruiting parents and sustaining their participation over time, which has also been experienced in previous studies of the ML program with parents of younger children. Additionally, there is a risk of spillover effects between study arms, as ML group leaders provide care and support to families in both the intervention and control arms.

The Co-Fam model has been co-designed with stakeholders to address the organizational gap between schools, primary care, and pediatric healthcare in the prevention and treatment of childhood overweight and obesity. If shown to be effective and feasible, the model has the potential to provide universal support to enhance health literacy while offering evidence-based treatment for affected children. By combining evaluation of both effectiveness and implementation, this study can generate valuable insights to inform policy and practice in settings that have similar healthcare structures as Sweden.

## Supplementary Information

Supplementary Material 1.

## Data Availability

No datasets were generated or analysed during the current study.

## Abbreviations

BMI: Body Mass Index
COM-B: Capability, Opportunity, Motivation and Behaviour
EPIS: The exploration, preparation, implementation, sustainment
ERIC: Expert Recommendations for Implementing Change
HCP: Healthcare Personnel
HSS: Healthy School Start
ICER: Incremental cost-effectiveness ratio
IOTF: International Obesity Task Force
IRLM: Implementation Research Logic Model
ML: More and Less
PC: Pediatric clinic
PHC: Primary Healthcare
QALY: Quality-Adjusted Life-Year
RCT: Randomized Controlled Trial
SPIRIT: Standard protocol items for clinical trials
TDF: Theoretical Domains Framework
TIDier: Template for intervention description and replication
T2D: Type 2 diabetes
